# Large Language Model Performance on Multistep Clinical Cases: Comparative Study Across Question and Case Levels

**DOI:** 10.2196/95342

**Published:** 2026-09-22

**Authors:** Jiaxue Cha, Yan Zhao, Hui Zong

**Affiliations:** 1 Centre for Medical Big Data and Artificial Intelligence The First Affiliated Hospital (Southwest Hospital) of Army Medical University (Third Military Medical University) Chongqing China; 2 School of Life Sciences and Technology Tongji University Shanghai China; 3 Department of Prevention Healthcare The First Affiliated Hospital (Southwest Hospital) of Army Medical University (Third Military Medical University) Chongqing China

**Keywords:** large language models, clinical cases, medical education, ChatGPT, DeepSeek

## Abstract

**Background:**

Most large language models (LLMs) have achieved passing scores on medical licensing examinations. However, most evaluations focus on single-question accuracy, overlooking performance on multistep patient management scenarios, such as making a diagnosis followed by a treatment plan. It is unclear if LLMs can maintain high performance on complete clinical cases.

**Objective:**

This study aimed to evaluate the performance of LLMs on multistep clinical cases and to investigate the impact of model size scaling and case complexity on performance stability.

**Methods:**

We curated a dataset of 189 unique clinical cases comprising 473 individual questions from the Chinese National Medical Licensing Examination. The number of questions related to each case ranged from 2 to 4. A physician with more than 8 years of clinical experience annotated the cases, confirming that 82.5% (156/189) contained sequentially dependent questions. We tested 4 representative LLMs: DeepSeek-R1, GPT-4o, Gemini-2.5-Flash, and Qwen2.5. We measured 2 metrics: the question pass rate (QPR) and the case pass rate (CPR), where a case was considered correct only if the model answered all its questions correctly. The consistency gap was calculated as the difference between QPR and CPR. Additionally, we tested Qwen2.5 at different parameter sizes (3B, 7B, 14B, and 32B) to examine the effect of model size. We also calculated the expected CPR and used the McNemar test with Bonferroni correction to compare model performance.

**Results:**

All LLMs achieved a QPR exceeding 83%. However, the CPR was lower for all models. DeepSeek-R1 achieved the highest QPR at 89.9% (425/473), with a CPR of 79.9% (151/189), corresponding to the smallest consistency gap at 10%. In contrast, GPT-4o exhibited a QPR of 83.5% (395/473) and a CPR of 65.6% (124/189), resulting in the largest consistency gap at 17.9%. DeepSeek-R1 and Qwen2.5-32B significantly outperformed GPT-4o in both QPR and CPR (*P*<.05). Observed and expected CPR values were closely aligned across models. Increasing the model size from 3B to 32B reduced the consistency gap by approximately 50%. As the number of questions per case increased, the consistency gap tended to increase across all evaluated LLMs.

**Conclusions:**

Current LLMs exhibit high accuracy in answering individual questions, but their ability to correctly answer all questions within a complete clinical case is substantially lower. This study design evaluated independent question answering, with responses aggregated at the case level. The performance depends heavily on model scale and case complexity. LLMs should be used as support tools rather than independent decision-makers in medical education and clinical practice.

## Introduction

Medical licensing examinations serve as the gold standard for assessing the professional competence of physicians, ensuring that they possess the requisite knowledge for safe clinical practice [1]. With the rapid evolution of AI, large language models (LLMs) have demonstrated remarkable capability in these standardized assessments [2]. Recent benchmarks indicate that state-of-the-art models, such as GPT-4 and other related LLMs, have achieved passing scores on the United States Medical Licensing Examination and the Chinese National Medical Licensing Examination (CNMLE), in some cases exceeding the performance of average human examinees [3-5]. These achievements have prompted growing interest in the potential application of LLMs as clinical decision support systems [6]. However, the current evaluation methodologies largely conceptualize clinical questions as isolated data points, focusing primarily on the accuracy of answering individual medical questions rather than performance on complete clinical cases.

In real-world clinical practice, physicians do not answer independent questions in isolation. They need to deal with complex, multistep clinical scenarios. A typical clinical encounter requires a logically structured sequence of decisions, progressing from symptom analysis and diagnostic testing to treatment planning and long-term follow-up [7]. In this context, accuracy metrics calculated solely on a per-question basis can be systematically overestimated [8]. For instance, a model might correctly diagnose a patient based on symptoms (question A) but subsequently recommend a clinically inappropriate or contraindicated treatment (question B) for the same patient. While a traditional per-item accuracy metric might score this as 50% accuracy, from a clinical perspective, this inconsistency renders the model unreliable and potentially unusable [9]. Therefore, the lack of assessment metrics focusing on the case-level performance, where success is defined by the correct resolution of the entire clinical trajectory, represents a significant methodological limitation in current AI medical research.

Prior studies have reported rapid improvements in performance of LLMs on the CNMLE, primarily focusing on question-level accuracy metrics. A synthesis of performance data from 8 independent studies published between 2023 and 2025 [10-17], collectively covering 28 distinct evaluation records on official CNMLE datasets (Multimedia Appendix 1), revealed a clear temporal pattern. Early-generation models, such as GPT-3.5, consistently failed to meet the passing threshold (60%), with accuracy scores ranging from 36.5% to 54.7%. In contrast, more recent models evaluated in 2024 and 2025, including GPT-4o, DeepSeek-R1, and Qwen3, generally achieved substantially higher performance, with several evaluations exceeding 90% accuracy. However, it is important to note that these published results predominantly rely on question-level accuracy measures, leaving the model performance on multistep clinical cases largely unexplored.

To address this gap between standardized testing scores and performance on multistep clinical tasks, this study uses multistep case-based questions from the CNMLE to systematically assess whether high question-level accuracy translates to success on complete clinical cases. We selected 4 representative models, including GPT-4o, Gemini-2.5-Flash, DeepSeek-R1, and Qwen2.5, to represent both internationally developed and domestically developed state-of-the-art architectures. Beyond comparing question-level pass rates with case-level pass rates, we further investigate the impact of model scaling on performance by analyzing the Qwen2.5 series across parameter sizes ranging from 3B to 32B. By shifting the focus from isolated accuracy measures to case-level outcome validity, this study aims to provide a more clinically grounded evaluation of the readiness of LLMs for integration into complex clinical workflows.

## Methods

### Data Collection

We collected a raw dataset from the CNMLE, covering a 5-year period from 2017 to 2021 [12]. The initial corpus consisted of 3000 multiple-choice questions (600 questions per year). These examinations are designed to assess the clinical competency of physicians and include both independent knowledge-based questions and case-based questions. To evaluate performance on complete clinical cases comprising multiple questions, our study focused exclusively on sequential case-based questions. These questions present a shared clinical vignette (eg, demographic characteristics, medical history, and symptoms), followed by a series of questions (ranging from 2 to 4 questions per case) that collectively cover different stages of clinical management (eg, from diagnosis to treatment).

We applied a rigorous 3-step screening process. First, we manually extracted all sequential case-based question sets from the raw dataset, resulting in 196 clinical cases comprising 494 individual questions. Second, we identified and removed 1 duplicate case (containing 4 questions) that appeared across different examination years, reducing the dataset to 195 cases with 490 questions. Third, to ensure the validity of the clinical context, we excluded cases lacking specific patient demographic or clinical information. This step removed 6 cases with 17 questions.

To clarify the extent to which subquestions within each case are interdependent, we invited a physician with more than 8 years of clinical experience to annotate all 189 cases. Each case was classified as having sequentially dependent questions if later questions (eg, about examinations or treatments) directly referred to the clinical context or decisions established in earlier questions (eg, a specific diagnosis). On the basis of this annotation, 82.5% (156/189) of cases were identified as containing such dependent question sequences. Representative examples of cases containing 2, 3, and 4 questions are presented in Multimedia Appendix 2.

### LLMs

To ensure a comprehensive evaluation of generative AI capabilities, we selected 4 representative LLMs that reflect the state of the art in both international and Chinese technological landscapes. The study included GPT-4o (OpenAI; released in May 2024) [18], Gemini-2.5-Flash (Google; released in June 2025) [19], DeepSeek-R1 (DeepSeek-AI Team; released in January 2025) [20], and Qwen2.5 (Alibaba Cloud; released in September 2024) [21]. These models were chosen for their widespread adoption and advanced linguistic proficiency.

Furthermore, to investigate the “scaling law,” specifically, how model parameter size influences case-level performance stability, we used the Qwen2.5 family across 4 distinct sizes: 3B, 7B, 14B, and 32B. This gradation allows a granular analysis of the relationship between computational scale and performance consistency across sequential questions.

The deployment methods varied according to model accessibility and architecture. We accessed GPT-4o, Gemini-2.5-Flash, and DeepSeek-R1 via their official APIs, whereas the Qwen2.5 series models were deployed locally using the Ollama framework. The specific API model identifiers were GPT-4o (gpt-4o), Gemini-2.5-Flash (gemini-2.5-flash), and DeepSeek-R1 (deepseek-reasoner). Qwen2.5 models (3B, 7B, 14B, and 32B parameters) were deployed locally using Ollama with the corresponding model tags (qwen2.5:3b, qwen2.5:7b, qwen2.5:14b, and qwen2.5:32b). The models were run using the default Ollama quantization configuration (Q4_K_M).

To rigorously test the models’ inherent knowledge and question-answering abilities without providing specific examples, a zero-shot prompting strategy was used for all queries. A unified prompt template was applied across all models (Multimedia Appendix 3). No manual tuning of decoding parameters was performed. Parameters were kept at the default settings provided by each model provider or inference framework. Each question was evaluated once. For clinical cases with multiple questions, individual questions were submitted independently in separate conversations without retaining conversation history from previous questions. These experiments were conducted in September 2025.

For the computational infrastructure, local inference was performed on a server running Ubuntu 22.04, using a hardware configuration that included an NVIDIA RTX 4090 (24 GB) graphics processing unit (GPU) and an NVIDIA Virtual 48 GB GPU to accommodate varying memory requirements. The software environment leveraged PyTorch (version 2.1), Ollama (version 0.9.0), and Transformers (version 4.25.4) to manage model loading and execution. The experimental workflows were managed through Jupyter Lab, and all model outputs were automatically logged into an SQL database for subsequent postprocessing and analysis.

### Metrics Definition

To evaluate the utility of LLMs in multistep clinical cases, we established a multidimensional metric framework that went beyond simple accuracy. We first calculated the question pass rate (QPR), which serves as the traditional baseline metric representing global accuracy. This indicator is calculated as follows: QPR=total number of correctly answered questions/total number of questions.

While QPR reflects general knowledge retrieval, it often fails to capture the holistic performance required for complex clinical scenarios where a single patient case involves multiple interdependent questions. To address this limitation, we introduced the case pass rate (CPR) as a stricter case-level accuracy metric. In this metric, a clinical case is considered successfully solved only if the model answers all subquestions associated with that specific case correctly; partial correctness is treated as a failure. Accordingly, CPR is calculated as follows: CPR=number of cases with all subquestions correct/total number of cases.

We estimated an expected CPR under the assumption that questions within a case are conditionally independent, using a stratified accuracy approach that does not rely on the correctness pattern of any individual case. Specifically, we first grouped all clinical cases by their length, that is, cases containing 2, 3, or 4 questions. For each length group, we calculated the overall question accuracy across all cases within that group: P_k_=correct questions in group k/total questions in group k.

This group-level accuracy reflects the model’s average performance on questions from cases of a given length, and crucially, it is estimated independently of the correctness pattern of any specific case. For any individual case, we then used the accuracy of its corresponding length group, rather than the case’s own accuracy, to estimate the expected probability that all questions in that case would be answered correctly under independence. The overall expected CPR was then obtained by averaging these case-specific expected probabilities across all 189 cases:



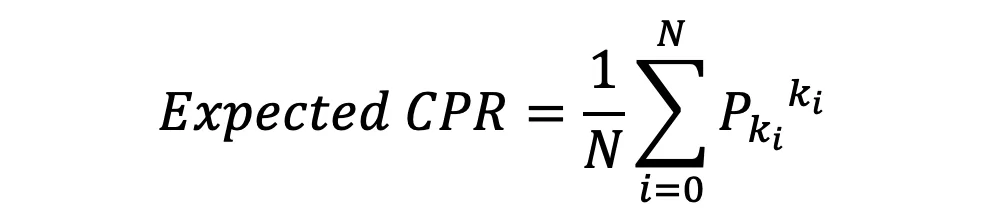



This stratified approach provides a valid independence-based benchmark for comparing against the observed CPR, free from the circularity of using a case’s own outcomes to predict its own performance.

Finally, to quantify the disparity between question-level and case-level performance, we computed the consistency gap. This metric represents the arithmetic difference between the QPR and CPR, defined as follows: consistency gap=QPR–CPR.

A smaller gap indicates better preservation of performance when moving from isolated questions to complete cases, whereas a larger gap suggests that the model’s ability to answer individual questions does not reliably translate to success on multistep case-level tasks. It is important to note that this gap is a descriptive measure of performance degradation from question-level to case-level aggregation, rather than a direct indicator of how consistently a model performs across dependent questions.

### Statistical Analysis

To assess whether the observed differences in QPR and CPR between models were statistically significant, we performed pairwise comparisons using the McNemar test. This test is appropriate for paired binary outcomes, as each question or case was evaluated by all models under identical conditions. For QPR comparisons, each of the 473 questions served as a paired unit; for CPR comparisons, each of the 189 cases served as a paired unit. Given that 6 pairwise comparisons were made across the 4 main models (DeepSeek-R1, Qwen2.5-32B, Gemini-2.5-Flash, and GPT-4o), we applied Bonferroni correction, with statistical significance defined as *P*<.05. For the Qwen2.5 scaling analysis, 6 pairwise comparisons were also made across the 4 parameter sizes (3B, 7B, 14B, and 32B), with the same significance threshold. All statistical analyses were performed using Python (version 3.13.13; Python Software Foundation) with the *statsmodels* library (version 0.14.6).

### Ethical Considerations

All data used in this study were obtained from publicly accessible sources. The research design did not include any intervention involving human participants or animals, and no sensitive or private information was collected or processed. Therefore, according to the ethical guidelines of the First Affiliated Hospital of Army Medical University, this study did not require ethical review or approval from the institutional ethics committee.

## Results

### Dataset Characteristics

The final curated dataset consisted of 189 unique clinical cases comprising 473 individual questions (Table 1). The structure of the 189 cases varied in complexity: 53.4% (n=101) cases contained 2 questions, 42.9% (n=81) cases contained 3 questions, and 3.7% (n=7) cases contained 4 questions. The dataset covers a diverse range of patient demographics representative of general medical practice. Among the patients described in the case vignettes, 48.7% (92/189) were male and 49.2% (93/189) were female. The minimum age of patients was 3 days, and the maximum age was 75 years. The clinical fields encompassed internal medicine, surgery, pediatrics, obstetrics and gynecology, and other specialties.

**Table 1:** Composition and characteristics of the curated clinical case dataset derived from the Chinese National Medical Licensing Examination (N=189).

Feature			Patients
Total questions, n			473
**Case structure (number of questions per case), n (%)**			
	2	101 (53.4)	
	3	81 (42.9)	
	4	7 (3.7)	
**Patient sex, n (%)**			
	Male	92 (48.7)	
	Female	93 (49.2)	
	Unspecified	4 (2.1)	
Patient age, range			3 days to 75 years

### Overall Performance

All 4 LLMs achieved QPR above 83% (Figure 1). DeepSeek-R1 showed the highest QPR at 89.9% (425/473), closely followed by Qwen2.5-32B at 89.6% (424/473), with no significant difference between them. DeepSeek-R1 significantly outperformed GPT-4o (395/473, 83.5%; *P*=.001) and Gemini-2.5-Flash (406/473, 85.8%; *P*=.02). Qwen2.5-32B also significantly outperformed GPT-4o (*P*=.003). Differences between Qwen2.5-32B and Gemini-2.5-Flash, as well as between Gemini-2.5-Flash and GPT-4o, were not significant. At the case level, DeepSeek-R1 achieved the highest CPR of 79.9% (151/189), significantly higher than GPT-4o (124/189, 65.6%; *P*<.001) and Gemini-2.5-Flash (135/189, 71.4%; *P*=.02). Qwen2.5-32B (144/189, 76.2%) also significantly outperformed GPT-4o (124/189, 65.6%; *P*=.03). No significant differences were found between DeepSeek-R1 and Qwen2.5-32B, Qwen2.5-32B and Gemini-2.5-Flash, or Gemini-2.5-Flash and GPT-4o. The consistency gaps (QPR minus CPR) were 10% for DeepSeek-R1, 13.5% for Qwen2.5-32B, 14.4% for Gemini-2.5-Flash, and 17.9% for GPT-4o. These results show that although all models performed well on individual questions, their performance drops consistently when handling complete cases, with GPT-4o showing the largest decline.

We further compared the observed CPR with the expected CPR for each model. The expected CPRs were 76.6% for DeepSeek-R1, 76.3% for Qwen2.5-32B, 68.6% for Gemini-2.5-Flash, and 64.4% for GPT-4o. When compared with the observed CPRs (DeepSeek-R1: 151/189, 79.9%; Qwen2.5-32B: 144/189, 76.2%; Gemini-2.5-Flash: 135/189, 71.4%; and GPT-4o: 124/189, 65.6%), the observed and expected values were closely aligned.

**Figure 1:**
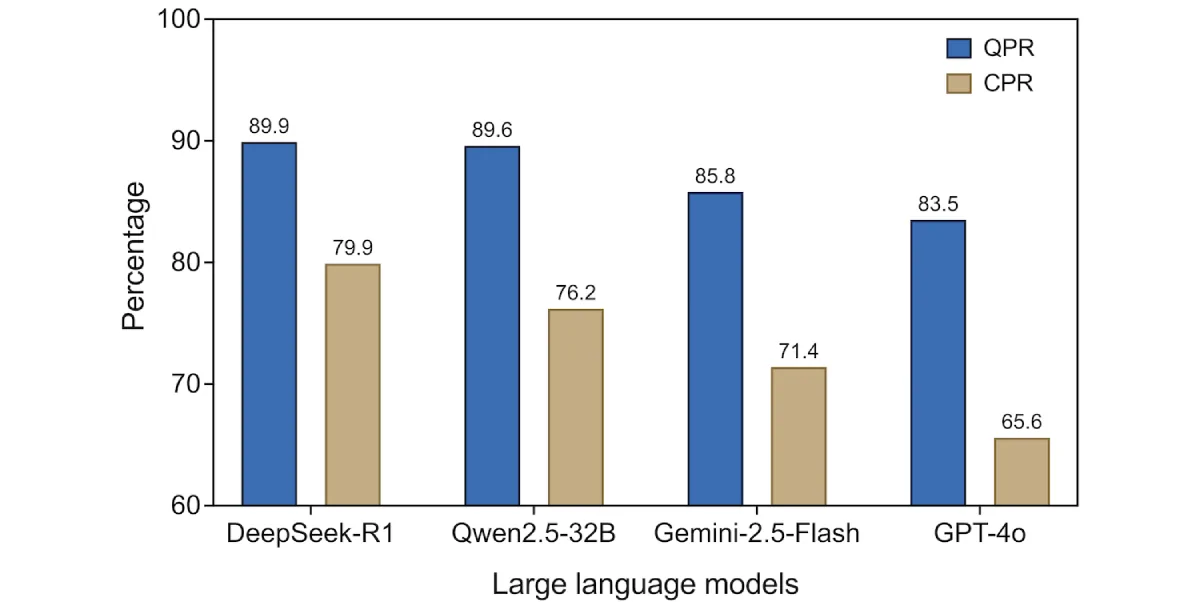
Comparative analysis of overall performance metrics across 4 large language models. The grouped bar chart displays the question pass rate (QPR) and case pass rate (CPR) for DeepSeek-R1, Qwen2.5-32B, Gemini-2.5-Flash, and GPT-4o. While all models achieved a high QPR exceeding 83%, a consistent performance degradation was observed in CPR, highlighting the challenge between isolated question answering and complete case-level clinical tasks.

### Performance Impact of Model Scaling

We observed a clear positive correlation between model parameter size and overall performance within the Qwen2.5 family. As illustrated in Figure 2, increasing the model size from 3B to 32B resulted in a consistent improvement in both metrics, with QPR increasing from 74.4% (352/473) to 89.6% (424/473) and CPR increasing from 48.1% (91/189) to 76.2% (144/189). All comparisons between the 3B model and larger models (7B, 14B, and 32B) showed significant differences (all *P*<.001). The 32B model also significantly outperformed the 7B model in QPR (*P*=.02), but differences between 7B vs 14B and 14B vs 32B in CPR were not significant. More importantly, the consistency gap showed a substantial decline as model size increased. The gap reduced by approximately 50%, from 26.3% in the 3B model to 13.5% in the 32B model. This pattern suggests that while smaller models may correctly answer individual questions, larger models are more likely to achieve success on complete patient cases.

**Figure 2:**
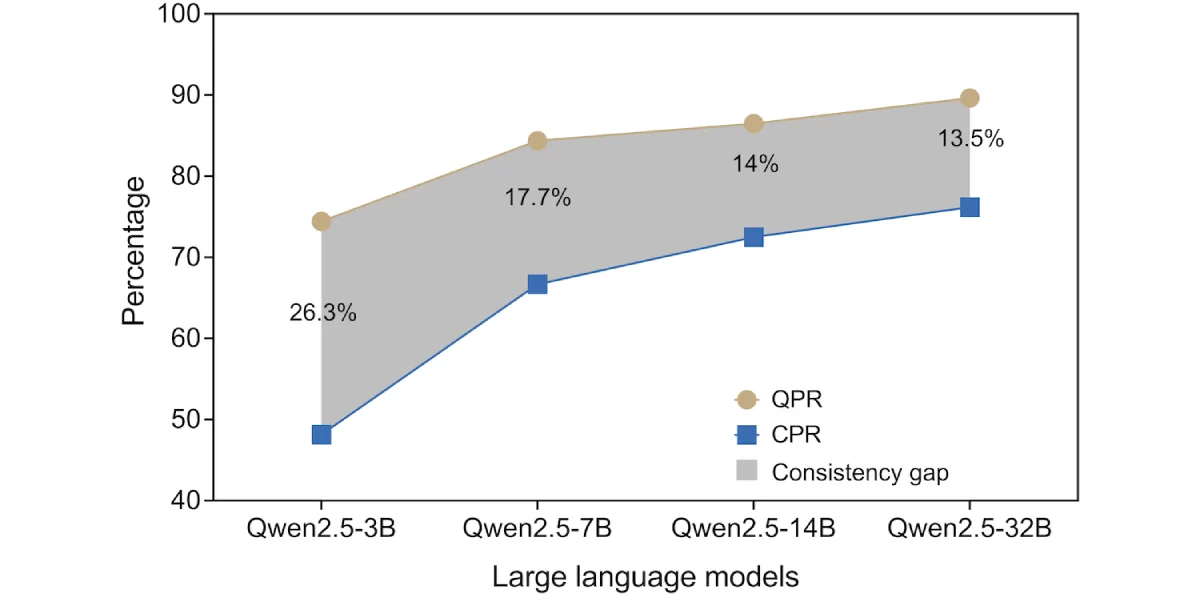
Impact of model scaling on performance across question and case levels. The line chart illustrates the performance of the Qwen2.5 series across different parameter sizes (3B, 7B, 14B, and 32B). The upper line represents the question pass rate (QPR), while the lower line indicates the case pass rate (CPR). The shaded region between the 2 curves highlights the consistency gap, which quantifies the disparity between question-level accuracy and case-level accuracy. As the model scale increases, both metrics improve, and the consistency gap decreases from 26.3% (3B) to 13.5% (32B), indicating performance degradation when moving from question-level accuracy to case-level aggregate accuracy.

### Impact of Case Complexity on Consistency Gap

To examine the relationship between task complexity and the consistency gap, we grouped the clinical cases according to the number of questions (2, 3, or 4 questions per case) and evaluated the consistency gap within each subgroup. As shown in Figure 3, the consistency gap increased with case complexity across all LLMs. This suggests that longer case sequences may be associated with greater performance degradation when moving from question-level to case-level evaluation. In lower-complexity settings (2 questions per case), the models showed similar levels of stability, with consistency gaps ranging from 7.9% (DeepSeek-R1 and Qwen2.5-32B) to 10.9% (GPT-4o). As complexity increased, however, the performance patterns differed. For cases with 3 questions, DeepSeek-R1 maintained a relatively small gap of 9.9%, whereas the gaps for Gemini-2.5-Flash and GPT-4o increased to 19.3% and 23.5%, respectively. The difference was largest in higher-complexity settings (4 questions per case). However, it is important to note that this subgroup contained only 7 cases, which limits the generalizability of this specific observation. DeepSeek-R1 limited the gap to 28.6%, while GPT-4o showed a substantial reduction in consistency, with the gap rising to 57.1%.

**Figure 3:**
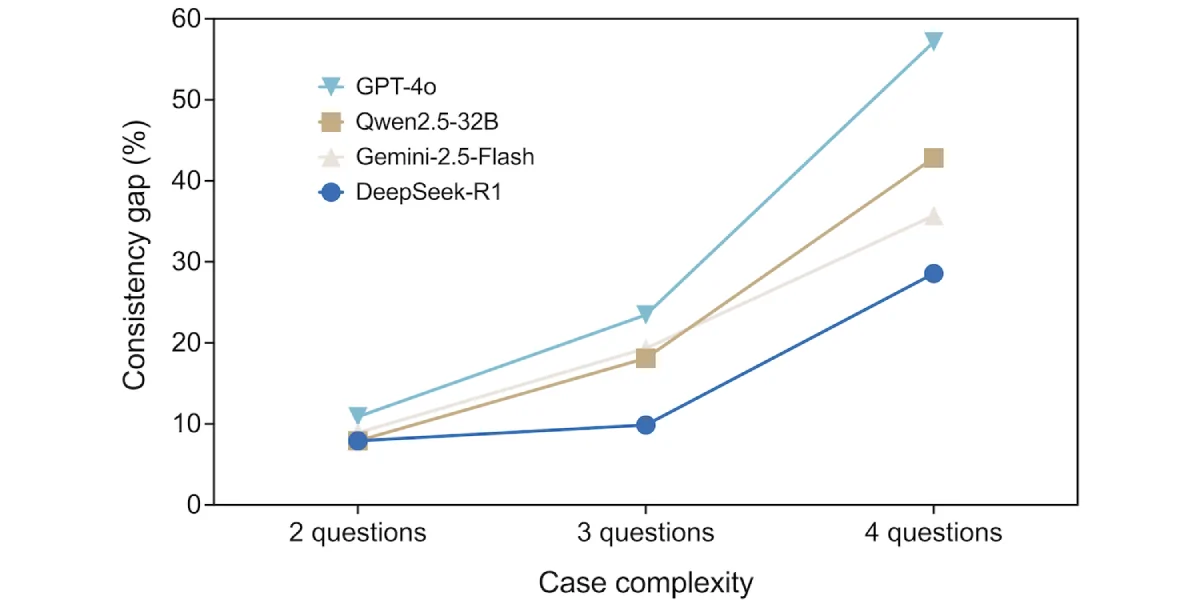
Impact of case complexity on consistency gap. The line chart shows changes in the consistency gap as the number of questions per clinical case increases from 2 to 4. A larger gap represents a greater difference between question-level accuracy and case-level accuracy. Across all models, the gap increases with case complexity.

## Discussion

### Principal Findings

This study provides a multidimensional evaluation of LLMs on the CNMLE, shifting the focus from traditional question-level accuracy to case-level performance on multistep clinical cases. Our results revealed a key finding. Although current models such as DeepSeek-R1 and GPT-4o achieved impressive question-level accuracy, their ability to solve complete clinical cases was significantly lower, revealing a consistency gap ranging from 10% to 17.9%. This indicates that current LLMs are excellent knowledge retrievers but show substantial performance decline when required to answer multiple interdependent questions correctly, highlighting a limitation in their practical applicability to complete clinical tasks. Furthermore, our model scaling analysis on the Qwen2.5 series (from 3B to 32B) demonstrated a strong negative correlation between model parameter size and the consistency gap. The consistency gap narrowed by nearly 50% as the model scaled up, suggesting that larger models are better at preserving performance when moving from question-level to case-level evaluation. Notably, our analysis of case complexity revealed a consistent pattern across models: the consistency gap between question-level and case-level performance tended to increase with the number of questions per case. As the number of questions within a clinical case increased, the consistency gap also tended to increase across the evaluated models, indicating greater performance degradation from question-level to case-level evaluation in longer cases.

### Comparison With Prior Work

Our results align with the general pattern reported in recent literature, confirming that state-of-the-art LLMs have largely surpassed the passing threshold for medical licensing examinations [2]. Previous studies have documented the impressive performance of models such as GPT-4 on the CNMLE, often citing accuracy rates exceeding 80% [15,17]. However, a critical limitation of these prior evaluations is their predominant focus on question-level accuracy. In these studies, clinical questions are typically treated as isolated data points. This approach simplifies the complexity of medical practice and may overestimate the reliability of LLMs in real-world settings.

In actual clinical practice, patient management is not a discrete event but rather a longitudinal process involving consecutive clinical actions [22]. A standard clinical workflow typically progresses from history taking and physical examination to the selection of diagnostic investigations, diagnostic confirmation, treatment strategy development, and subsequent prognosis assessment or follow-up planning [23,24]. These steps are intrinsically linked. A correct diagnosis is clinically insufficient if followed by an inappropriate or incompatible treatment recommendation. Therefore, evaluating models based on a single-item accuracy metric fails to capture the case-level continuity required for safe and reliable medical decision-making.

Our study addresses this methodological gap. By leveraging the case-based questions from the CNMLE, we evaluated model performance using groups of questions that represent different stages of clinical management within a shared patient vignette. In our assessment, a clinical case consisted of multiple logically interdependent questions covering prevention, examination, diagnosis, treatment, and rehabilitation. We propose the CPR, which defines success only when the model answers all related questions for a given patient correctly. This shift from question-level performance measures to a case-level evaluation paradigm provides a more stringent and meaningful benchmark, thereby revealing limitations of current LLMs that are masked by high accuracy scores in traditional assessments.

### Educational Implications

This study makes 3 main contributions for application of LLMs in medical education. First, while prior evaluations of LLMs on medical licensing examinations have focused almost exclusively on question-level accuracy [25], we introduced a case-level evaluation framework that better reflects the multistep nature of clinical cases. By requiring all questions within a case to be answered correctly, the CPR provides a more stringent and clinically meaningful benchmark. Second, we empirically demonstrated a substantial gap between question-level and case-level performance across multiple state-of-the-art models, showing that high accuracy on isolated questions did not reliably translate to success on complete clinical cases. Third, we quantified the effect of model scaling and case complexity on this gap, revealing that while larger models perform better, the performance degradation from multistep cases persisted across all model sizes.

This study has important implications for how LLMs should be integrated into medical education. There is growing interest in using LLMs as virtual teaching tools for medical students [26-29]. However, our results indicate that such use requires caution. First, model size had a clear impact on performance. Smaller LLMs, such as those with fewer than 7B parameters, showed large consistency gaps and low CPR. As a result, they are not suitable for independent teaching tasks. These models may encourage fragmented learning, where students learn to answer individual questions correctly but fail to understand the overall logic of patient management. Second, with respect to overreliance on AI, students need to be made aware of the false sense of understanding that AI systems may create [30]. Even more advanced models, such as GPT-4o, may answer some questions within a clinical case incorrectly despite strong overall question-level performance. Medical education programs should therefore train students to critically evaluate AI-generated answers across the full clinical context rather than accepting individual answers without verification. Overall, the use of LLMs in medical education should be carefully guided [31]. AI systems should be viewed not as authoritative instructors but as support tools whose outputs require continuous human review and judgment.

### Future Directions

Future research should focus on reducing the consistency gap identified in this study. From a methodological perspective, improving prompting strategies may help models achieve more stable performance in multistep clinical cases. For example, future studies could explicitly preserve conversation history across questions and evaluate sequential prompting strategies, including step-by-step prompts, to determine whether such approaches improve performance across successive stages of a case. In addition, real-world clinical information is not limited to text alone. Future evaluations should therefore include medical images and clinical records to better reflect actual clinical settings. Testing models with radiology images and narrative medical notes may provide a more realistic assessment of clinical performance. Finally, training open-source LLMs on well-curated clinical case data may further improve their performance. LLMs could benefit from task-specific training focused on clinical workflows. This approach may support the development of specialized medical systems that perform more reliably than general-purpose models in complex clinical tasks.

### Limitations

Our study has several limitations. First, the evaluation was limited to text-based questions. Cases requiring interpretation of medical images, such as electrocardiograms or radiographs, were not included, even though these are essential components of routine clinical practice. Second, our analysis of case complexity was constrained by an imbalanced dataset. Most cases contained 2 or 3 questions, while only a few cases had 4 questions. Consequently, the observed performance drop in the most complex cases should be viewed as a preliminary finding. The generalizability of this result needs to be validated with a more balanced set of clinical cases. Third, the study was conducted only in Chinese. While this offers useful non-English evidence for medical AI research, the applicability of the results to other languages or medical systems requires further validation. Finally, although official licensing examination questions were used, the multiple-choice format does not fully reflect the complexity of real-world clinical practice. The CPR measures complete correctness across all questions within a case, but it does not directly assess whether answers are logically compatible with one another. In real practice, clinicians face open-ended problems with many possible actions, rather than a fixed set of predefined options. Additionally, because each question was submitted independently without retaining prior conversation history, our results reflect performance on independently answered questions aggregated at the case level rather than true sequential performance across clinical steps.

### Conclusions

This study provides a critical reassessment of LLMs in CNMLE by distinguishing between question-level performance and case-level performance. We found that while state-of-the-art models, such as DeepSeek-R1 and GPT-4o, demonstrated exceptional knowledge retrieval with high QPR, they exhibited a lower performance in CPR when navigating multistep clinical scenarios. This consistency gap indicates that current models frequently fail to achieve the same level of success on complete clinical cases as they do on individual questions, highlighting a practical limitation in their application to multistep clinical tasks. Furthermore, our analysis confirmed that while increasing model scale reduced the consistency gap, the challenge of handling complex cases remained a universal bottleneck. Consequently, we conclude that traditional single-question benchmarks overestimate the clinical capabilities of LLMs. Future research should further investigate model performance using explicitly sequential clinical evaluation designs. Until LLMs can reliably handle complete clinical cases with the same proficiency as individual questions, they should be deployed as support assistants in medical education and health care environments.

## Appendix

Multimedia Appendix 1 Summary of large language model performance on the Chinese National Medical Licensing Examination from 8 peer‑reviewed studies.

Multimedia Appendix 2 Representative examples of 2-, 3-, and 4-part clinical cases with annotated question dependency structure.

Multimedia Appendix 3 Zero-shot prompting template used for large language model evaluation.

## Abbreviations

CNMLE: Chinese National Medical Licensing Examination
CPR: case pass rate
GPU: graphics processing unit
LLM: large language model
QPR: question pass rate

## Data Availability

The data that support the findings of this study are available from the corresponding author on reasonable request.
