# Supplementary material for: Large Language Model Performance on Multistep Clinical Cases: Comparative Study Across Question and Case Levels
Source: JMIR Med Educ. 2026 Sep 22;12:e95342. doi: 10.2196/95342 (PMC13643263; doi:10.2196/95342)
Supplement: Supplementary file 1 [file mededu_v12i1e95342_app1.docx]

**Multimedia Appendix 1.** Summary of large language model performance on the Chinese National Medical Licensing Examination from 8 peer‑reviewed studies.

| PMID | Exam_year | Models | Model_release_date | Accuracy |
| --- | --- | --- | --- | --- |
| 41237388 | 2021 | ChatGPT-o1 pro | 2024-12 | 75.00% |
|  | 2021 | DeepSeek-V3 | 2024-12 | 93.00% |
|  | 2021 | GPT-4o | 2024-5 | 75.30% |
|  | 2021 | ChatGPT-o3 mini | 2025-1 | 75.80% |
|  | 2021 | DeepSeek-R1 | 2025-1 | 96.00% |
|  | 2021 | Qwen3 | 2025-4 | 92.50% |
|  | 2021 | ERNIE 4.5 Turbo | 2025-4 | 95.30% |
| 40459679 | 2024 | GPT-4o | 2024-5 | 92.00% |
|  | 2024 | DeepSeek-R1 | 2025-1 | 87.20% |
| 40269046 | 2020 | GPT-3.5 | 2022-11 | 50.50% |
|  | 2021 | GPT-3.5 | 2022-11 | 50.80% |
|  | 2021 | GPT-4 | 2023-3 | 73.20% |
|  | 2020 | GPT-4 | 2023-3 | 74.70% |
|  | 2020 | GPT-4o | 2024-5 | 84.20% |
|  | 2021 | GPT-4o | 2024-5 | 88.20% |
| 39697797 | 2023 | ERNIE | 2023-3 | 61.70% |
|  | 2023 | GPT-4 | 2023-3 | 65.20% |
| 39140269 | 2022 | GPT-3.5 | 2022-11 | 54.00% |
|  | 2022 | GPT-4 | 2023-3 | 72.70% |
| 38355517 | 2020 | GPT-3.5 | 2022-11 | 51.17% |
|  | 2019 | GPT-3.5 | 2022-11 | 51.26% |
|  | 2017 | GPT-3.5 | 2022-11 | 53.50% |
|  | 2018 | GPT-3.5 | 2022-11 | 54.67% |
|  | 2021 | GPT-3.5 | 2022-11 | 54.67% |
| 38039286 | 2022 | GPT-4 | 2023-3 | 73.67% |
| 37581690 | 2022 | GPT-3.5 | 2022-11 | 36.50% |
|  | 2021 | GPT-3.5 | 2022-11 | 45.80% |
|  | 2020 | GPT-3.5 | 2022-11 | 47.00% |
