# Supplementary material for: Large Language Model Performance on Multistep Clinical Cases: Comparative Study Across Question and Case Levels
Source: JMIR Med Educ. 2026 Sep 22;12:e95342. doi: 10.2196/95342 (PMC13643263; doi:10.2196/95342)
Supplement: Supplementary file 2 [file mededu_v12i1e95342_app2.docx]

**Multimedia Appendix 2.** Representative examples of 2-, 3-, and 4-part clinical cases with annotated question dependency structure.

This appendix provides three representative examples of sequential case-based questions from the Chinese National Medical Licensing Examination (CNMLE), illustrating the dependency relationships between sub-questions within each clinical case. The annotations were performed by a physician with over 8 years of clinical experience. Cases were classified as containing sequentially dependent questions if later questions (e.g., about examinations or treatments) directly referred to the clinical reasoning established in earlier questions (e.g., a specific diagnosis).

**Example 1: 2-Part Case.**

**Rheumatoid Arthritis Diagnosis and Treatment**

**Case vignette**: A 45-year-old female presents with a 5-month history of swelling and pain in the bilateral proximal interphalangeal joints, wrists, and ankles. Physical examination reveals fusiform swelling and tenderness of the bilateral proximal interphalangeal joints. Laboratory findings: ESR 45 mm/h, positive RF, and positive anti-cyclic citrullinated peptide (anti-CCP) antibody.

**Question 1 (Diagnosis)**:

What is the most likely diagnosis?

A. Osteoarthritis

B. Rheumatoid arthritis (Correct)

C. Gouty arthritis

D. Spondyloarthritis

E. Systemic lupus erythematosus

**Question 2 (Treatment):**

What is the first-line medication to control disease progression?

A. Methotrexate (Correct)

B. Ibuprofen

C. Aspirin

D. Cyclophosphamide

E. Prednisone

**Dependency annotation**: Question 2 directly depends on Question 1. The diagnosis of rheumatoid arthritis (Q1) determines that methotrexate, a disease-modifying antirheumatic drug (DMARD), is the appropriate first-line treatment. If the model incorrectly answers Q1 (e.g., choosing osteoarthritis or gout), it would likely select an inappropriate treatment for Q2, as the treatment strategy is diagnosis-specific.

**Example 2: 3-Part Case.**

**Cholelithiasis Diagnosis, Investigation, and Management**

**Case vignette**: A 30-year-old female with a 2-year history of gallbladder stones found on routine examination. Recently, she experienced persistent upper abdominal pain radiating to the right shoulder after consuming fatty foods, accompanied by nausea and vomiting, with spontaneous relief after rest and recurrent episodes. Physical examination: marked upper abdominal tenderness, suspected positive Murphy's sign, no costovertebral angle tenderness or percussion pain.

**Question 1 (Diagnosis):**

What is the initial diagnosis to consider?

A. Cholelithiasis (gallbladder stones) (Correct)

B. Nephrolithiasis

C. Gastric ulcer

D. Intestinal obstruction

E. Acute exacerbation of chronic pancreatitis

**Question 2 (Investigation):**

What is the first-choice examination to confirm the diagnosis?

A. MRI

B. Urinalysis

C. Abdominal ultrasound (Correct)

D. Serum amylase

E. Barium meal X-ray

**Question 3 (Management):**

If the diagnosis is confirmed, what is the treatment plan?

A. Fasting, continuous gastrointestinal decompression, and somatostatin infusion

B. Extracorporeal shock wave lithotripsy

C. Antacid and anti-inflammatory therapy

D. Laparoscopic cholecystectomy (Correct)

E. Laxative enema

**Dependency annotation:** Question 2 depends on Question 1. Abdominal ultrasound is the preferred imaging modality to confirm gallstones. Question 3 depends on both Question 1 and Question 2, once the diagnosis of cholelithiasis is confirmed, laparoscopic cholecystectomy is the standard surgical treatment. An incorrect answer to Q1 (e.g., nephrolithiasis or pancreatitis) would lead to inappropriate investigations and management plans for subsequent questions.

**Example 3: 4-Part Case.**

**Cervical Spondylosis Diagnosis, Differential Diagnosis, Surgery, and Etiology**

**Case vignette:** A 55-year-old male presents with a 6-month history of bilateral lower limb weakness, more pronounced on the right side, with unstable gait for the past 2 months and inability to button his shirt with the right hand. No history of trauma or fever. Physical examination: no significant cervical tenderness; decreased sensation in the bilateral forearms, hands, and ulnar aspects of the upper arms, more pronounced on the right; increased muscle tone in all four limbs; hyperactive biceps reflex, hyperactive bilateral knee and ankle reflexes; right patellar clonus positive; right Babinski sign positive.

**Question 1 (Diagnosis):**

What is the most likely diagnosis?

A. Cerebral stroke

B. Cervical spondylosis (Correct)

C. Cervical tumor

D. Cervical tuberculosis

E. Cervical nerve root neuritis

**Question 2 (Differential Diagnosis):**

Which ancillary examination is most helpful for differential diagnosis?

A. Cervical X-ray

B. Cervical tomography

C. Electromyography

D. Cervical MRI (Correct)

E. Radionuclide scanning

**Question 3 (Management):**

What treatment should be considered?

A. Cervical traction with head-halter

B. Hormone therapy

C. Massage therapy

D. Surgery (Correct)

E. Cervical collar

**Question 4 (Etiology):**

What is the causative factor?

A. Viral infection

B. Bacterial infection

C. Degenerative changes (Correct)

D. Allergic reaction

E. Hypertension

**Dependency annotation:** Question 2 depends on Question 1. Cervical MRI is the most useful imaging modality to confirm cervical spondylosis and rule out other conditions such as tumor or tuberculosis. Question 3 depends on Questions 1 and 2, surgical intervention is indicated for cervical spondylosis with significant neurological deficits. Question 4 relates back to Question 1, the underlying etiology of cervical spondylosis is degenerative changes. An incorrect answer to Q1 would lead to inappropriate choices for Q2, Q3, and Q4, as each subsequent question is tailored to the specific diagnosis.
