# Supplementary material for: Large Language Model Performance on Multistep Clinical Cases: Comparative Study Across Question and Case Levels
Source: JMIR Med Educ. 2026 Sep 22;12:e95342. doi: 10.2196/95342 (PMC13643263; doi:10.2196/95342)
Supplement: Supplementary file 3 [file mededu_v12i1e95342_app3.docx]

**Multimedia Appendix 3.** Zero-shot prompting template used for large language model evaluation.

The original prompt was written in Chinese. The following content is an English translation provided for readability.

Prompt template:

*Please select the most appropriate answer from the five options for the following medical multiple-choice question and output the answer in JSON format as follows:*

*```json*

*{*

*"answer": "X"*

*}*

*```*

*where X represents one of the options A, B, C, D, or E. Please do not provide any explanation or additional information.*

*Question:*

*{question}*

*A. {option A}*

*B. {option B}*

*C. {option C}*

*D. {option D}*

*E. {option E}*
